# Vertical Transmission of SARS-CoV-2 Infection and Miscarriage in the Second Trimester: Report of an Immunohistochemically Proven Case

**DOI:** 10.3390/clinpract12040061

**Published:** 2022-07-26

**Authors:** Dimitar Metodiev, Margarita Ruseva, Dimitar Parvanov, Rumiana Ganeva, Maria Handzhiyska, Nina Vidolova, Georgi Stamenov

**Affiliations:** 1 Department of Clinical Pathology, Nadezhda Women’s Health Hospital, 1373 Sofia, Bulgaria; 2 Neuropathological Laboratory, University Hospital “Saint Ivan Rilski”, 1431 Sofia, Bulgaria; 3 Department of Research, Nadezhda Women’s Health Hospital, 1373 Sofia, Bulgaria; dimparvanov@abv.bg (D.P.); rum.ganeva@gmail.com (R.G.); mariavh@abv.bg (M.H.); nina.vidolova@abv.bg (N.V.); 4 Department of Obstetrics & Gynaecology, Nadezhda Women’s Health Hospital, 1373 Sofia, Bulgaria; g.stamenov1@abv.bg

**Keywords:** SARS-CoV-2, placental tropism, vertical transmission, nucleocapsid protein

## Abstract

It is an acknowledged fact that SARS-CoV-2 exhibits tropism for the human placenta. A possible mechanism of SARS-CoV-2 entry into host cells is via angiotensin-converting enzyme 2 (ACE2) receptors, which are expressed in trophoblasts, endothelial cells, and macrophages. The present study describes a case of spontaneous miscarriage in the 20th gestational week after maternal SARS-CoV-2 infection. The placenta and various fetal organs were examined for structural alterations and expression of the viral nucleocapsid protein and several immune cell markers via immunohistochemistry (IHC). Histopathological examination of the placenta revealed acute chorioamnionitis, acute subamnionic placentitis, multiple intervillous thrombi, increased fibrinoid deposition, and necrotic changes of the chorionic villi. Immunohistochemistry confirmed the presence of SARS-CoV-2 nucleocapsid protein regions predominantly in the syncytiotrophoblast. Staining of the placental tissue for different markers helped elucidate the distribution of immune cells. Pathomorphological examination of the fetal organs demonstrated changes in microcirculation with the presence of sludge phenomenon and diapedesis haemorrhages, mostly in the lungs, brain, and myocardium. IHC staining of fetal organs revealed expression of SARS-CoV-2 nucleocapsid protein, which was detected to the highest extent in the brain, lungs, and liver. The findings of the present report support the hypothesis of possible vertical transmission of SARS-CoV-2 from mother to fetus.

## 1. Introduction

Viral infections frequently cause pregnancy complications. Some viruses are associated with mild maternal morbidity and are not detrimental to the developing fetus. Nonetheless, others such as the cytomegalovirus have a mild or asymptomatic course in pregnant women, yet often cause congenital anomalies [1].

Viruses can spread to the unborn child through the decidua and placenta via ascending infections of the lower reproductive organs, as is the case with colpitis and cervicitis. In other cases, the viral infection enters the chorionic villi from the maternal bloodstream, and consequently spreads in the fetus via the villous vasculature [2].

Data on the impact of in utero SARS-CoV-2 infection during the first and second trimesters are inconclusive, and information obtained during the third is limited. Nevertheless, there have been reports on newborns with respiratory distress syndrome or stillbirths after maternal COVID-19 infection during the third trimester, which indicates possible transplacental transmission of the SARS-CoV-2 virus [3,4,5,6].

The aim of the present report was to investigate the placenta and various fetal organs for structural alterations and expression of SARS-CoV-2 nucleocapsid protein and several immune cell markers after miscarriage following maternal SARS-CoV-2 infection in the second trimester.

## 2. Case Report

A 33-year-old pregnant patient (gravida 2, para 1) presented with rhinitis, headache, arthralgias, and sub-febrile temperature to our department at 19 weeks of gestation. She tested positive for SARS-CoV-2 by RT-PCR on a nasopharyngeal swab. At that moment, her blood pressure and heart rate were normal and oxygen saturation was 98%. Laboratory findings supported the diagnosis, since the patient showed an increased number of neutrophils, decreased lymphocyte count, and slightly elevated C-reactive protein. Her chest X-ray showed no abnormalities.

After a week, the minor COVID-19 symptoms had disappeared. The patient experienced abdominal and lower back pain, as well as severe uterine contractions. Physical examination did not reveal any signs of pneumonia. Vaginal swab and urine sample were collected for microbiological investigation. Cultures from both were negative for common pathogens (bacteria, fungi). Normal fetal morphology, growth, and amniotic fluid were detected using an ultrasound scan. Afterwards, the fetus showed heart rate decelerations. A stillborn infant was delivered after unstoppable labor at 20 weeks gestation. A fetal autopsy was performed. The fetus was 24 cm in length and weighed 254 g. The external habitus did not exhibit general edema, congestive appearance, or congenital malformations. Internal examination revealed no structural abnormalities. Postmortem fetal samples of lungs, heart, thymus, brain, spinal cord, kidneys, suprarenal glands, liver, spleen, stomach, pancreas, testes, epididymis, and bowels were collected for histological and additional immunohistochemistry (IHC) studies.

**Figure 1:**
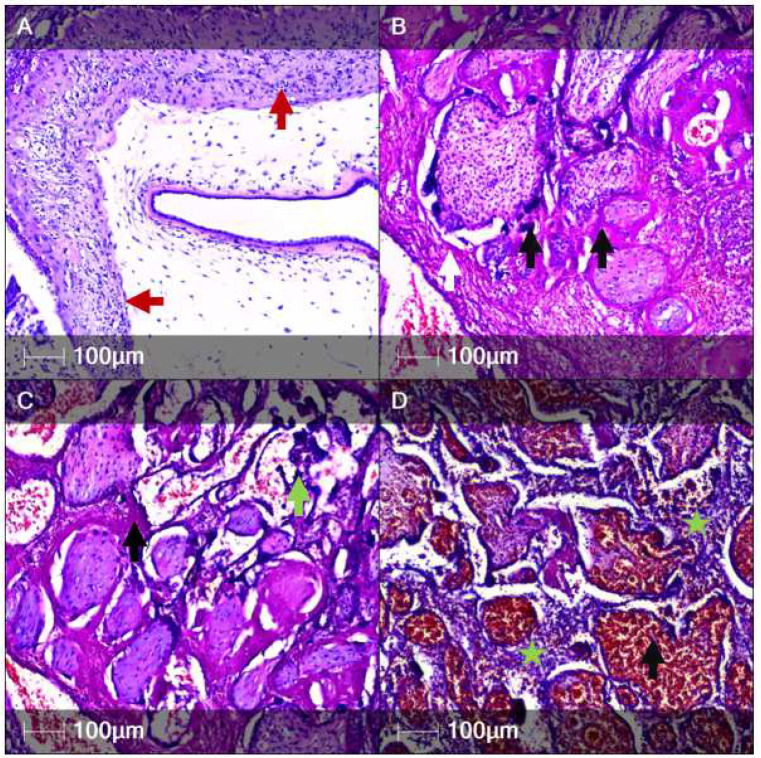
Histological examination of the placenta (H&E staining). The panel depicts various components and pathological features of the placenta. (**A**) Acute chorioamnionitis with signs of necrosis and leukocytoclasis (red arrows); (**B**) Subamniotic placentitis with intervillous thrombi (white arrow) and necrotic syncytiotrophoblasts (black arrows); (**C**) Syncytiotrophoblast necrosis (dark blue, green arrow) and intevillous cell debris (black arrow); (**D**) Subamniotic placentitis with predominantly intervillous neutrophil infiltrates (intervillositis—green asterisk) and severe intravillous congestion and haemorrhages (black arrow).

One of our main focus points was examination of the placenta. The placental weight was 50 g, with measurements 12.5 × 8 × 2 cm. Histological examination via hematoxylin and eosin (H&E) staining revealed acute chorioamnionitis (Figure 1A), acute subamnionic placentitis (Figure 1B), multiple intervillous thrombi, chronic deciduitis, perivillous fibrin deposition with syncytiotrophoblast necrosis (Figure 1C), chronic intervillositis and villitis with signs of inflammatory activity (presence of neutrophils), and villous hyperaemia and haemorrhages (Figure 1D).

**Figure 2:**
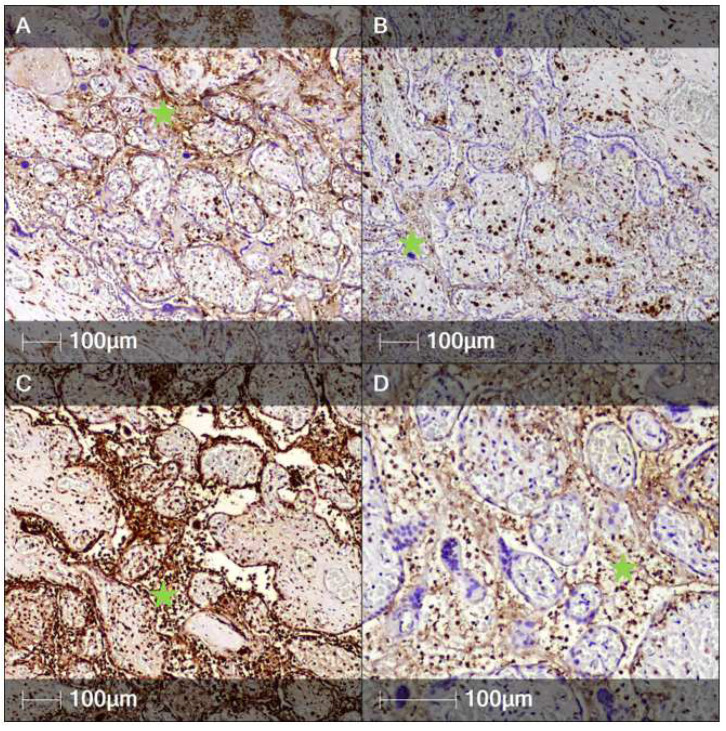
Immunohistochemistry staining of intervillous spaces. Representative images of several immune cell markers expressed in intervillous spaces (asterisks). (**A**) CD14 staining (monocytes); (**B**) CD68 staining (macrophages); (**C**) Neutrophil elastase staining (neutrophils); (**D**) CD79a staining (B-lymphocytes).

**Figure 3:**
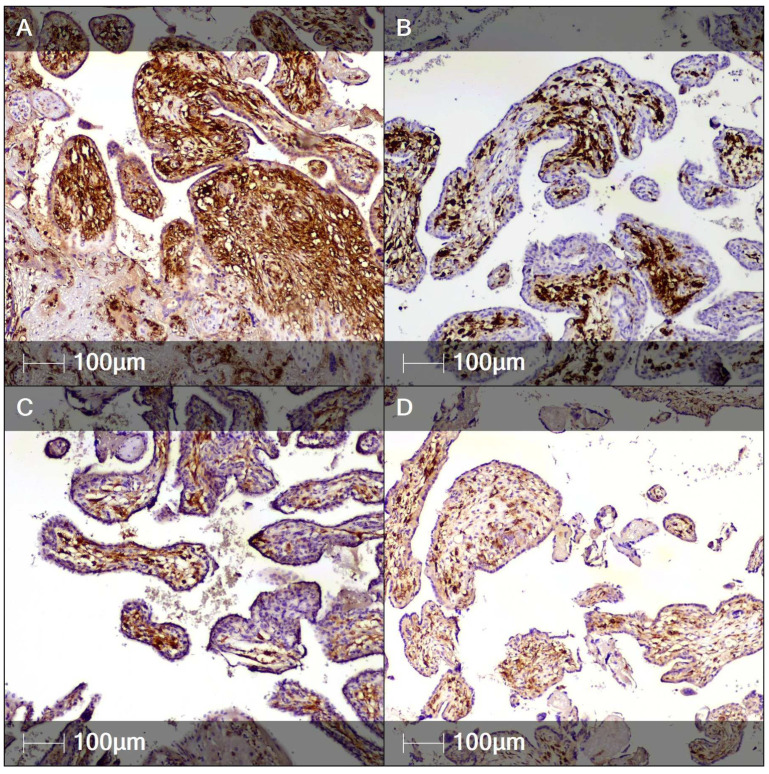
Immunohistochemistry staining of chorionic villi. Representative images of several immune cell markers expressed in the chorionic villi. (**A**) CD14 staining (monocytes); (**B**) CD68 staining (macrophages); (**C**) CD4 staining (T-helper lymphocytes); (**D**) CD8 staining (cytotoxic T-lymphocytes).

Heterogeneous inflammatory infiltrates in the intervillous areas of the placenta consisted of numerous CD14- and CD68-positive macrophages (Figure 2A,B). A large number of neutrophil leukocytes were visualized via anti-neutrophil elastase antibody (Figure 2C). B-lymphocytes, which constituted a significant component of the inflammatory population, were identified by the marker CD79a (Figure 2C). Additionally, IHC markers CD4 and CD8 allowed assessment of the spatial distribution of the remaining immune cells, namely T-lymphocytes, involved in the inflammatory processes of the placenta and its membranes. The villous stroma predominantly consisted of macrophages positive for CD14 and CD68 and fewer CD4- and CD8-positive T-lymphocytes and neutrophil leukocytes (Figure 3).

**Figure 4:**
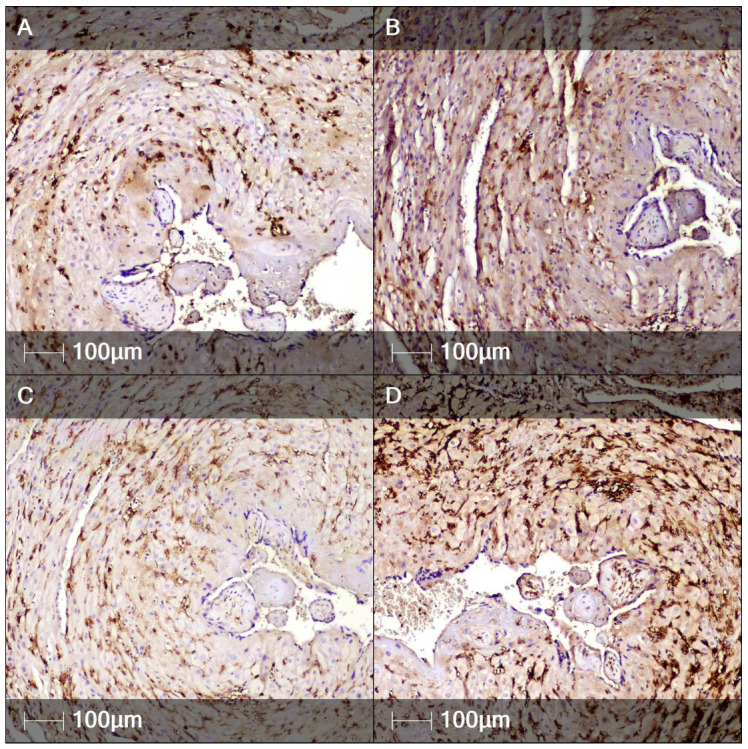
Immunohistochemistry staining of decidua. Representative images of several immune cell markers expressed in the decidua (IHC). (**A**) CD56 staining (natural killer cells); (**B**) CD4 staining (T-helper lymphocytes); (**C**) CD8 staining (cytotoxic T lymphocytes); (**D**) CD14 staining (monocytes).

Occasionally, the inflammatory infiltrates affected the villous surface, with signs of syncytiotrophoblast necrosis and cell debris—eosinophilic material and fibrin fibers in the narrowed intervillous spaces. Of note was the large number of CD56-positive NK cells and CD4-, CD8-, and CD14-positive cells in the decidua, which constitute a histological hallmark of chronic deciduitis (Figure 4).

**Figure 5:**
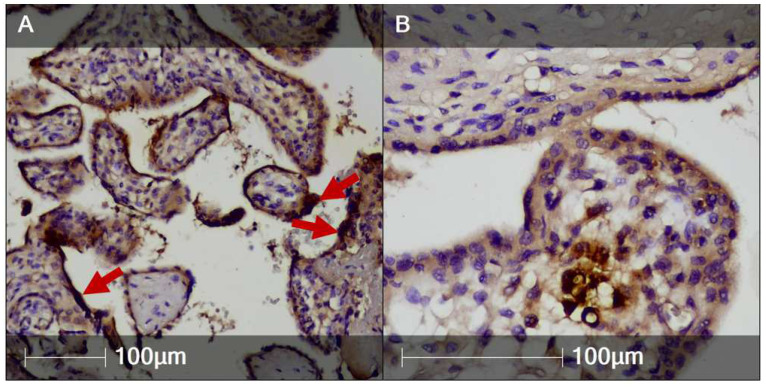
SARS-CoV-2 nucleocapsid protein in the inflamed placental tissue (IHC)**.** Areas of the placenta positively stained for SARS-CoV-2 nucleocapsid protein. (**A**) Syncytiotrophoblast (red arrows); (**B**) Villous stromal cells.

Notably, the presence of SARS-CoV-2 nucleocapsid protein in the inflamed placental tissue was confirmed via IHC. It was predominantly found in syncytiotrophoblast cells, but also on occasion in villous stromal cells—possibly fetal monocytes–Hofbauer cells (Figure 5). The umbilical cord and placental membranes were negative for SARS-CoV-2 nucleocapsid protein.

In terms of morphological presentation, the autopsy revealed that the internal organs of the fetus exhibited immature immune response, which is characteristic for inflammatory events in such early gestation.

**Figure 6:**
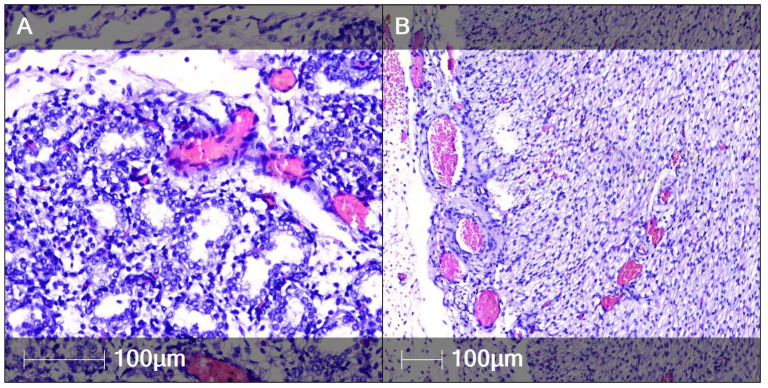
Vascular anomalies in fetal tissue (H&E staining). Vascular congestion with erythrocyte aggregation (pink staining) in fetal organs. (**A**) Lung; (**B**) Myocardium.

**Figure 7:**
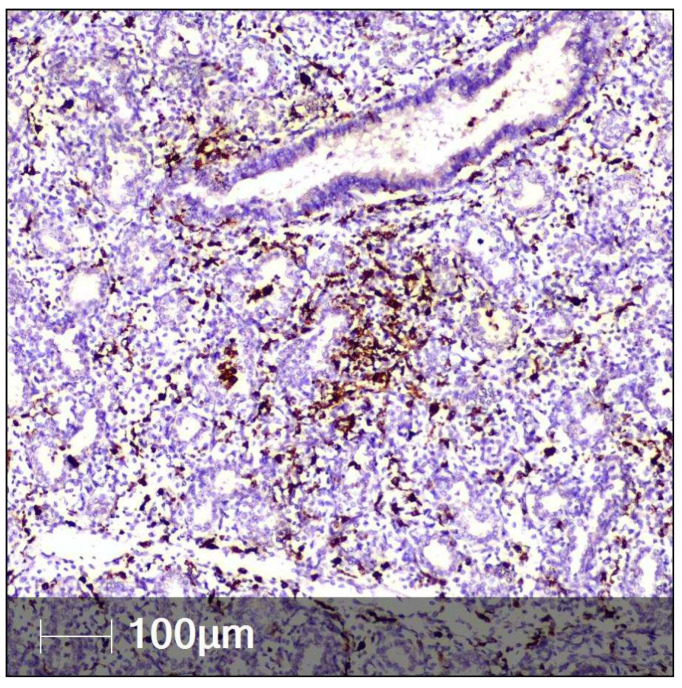
CD68-positive macrophages in fetal lung tissue (IHC).

Both lungs were in the pseudoglandular stage. Circulatory anomalies were observed, namely vascular congestion with erythrocyte aggregation with sludge phenomenon in the microvasculature and occasional diapedesis haemorrhages (Figure 6A). There was hypercellularity composed of CD68-positive infiltrating macrophages. These were found in the interstitium, including in the subpleural areas and in peribronchiolar areas, with the tendency to form CD68-positive nodules (Figure 7).

Similar circulatory abnormalities to those in the lungs were observed in the microcirculatory bed of other internal organs, particularly in the myocardium (Figure 6B).

**Figure 8:**
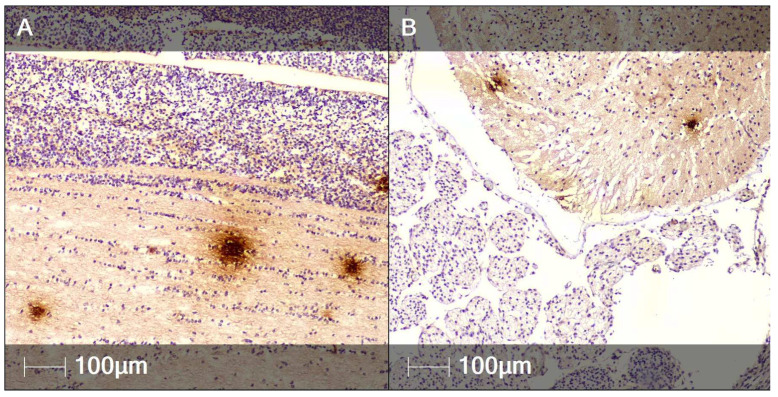

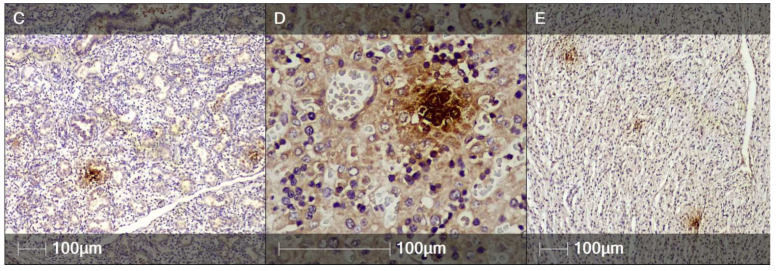
SARS-CoV-2 nucleocapsid protein in several fetal organs (IHC). Representative images of various fetal tissues positively stained for SARS-CoV-2 nucleocapsid protein. (**A**) Brain; (**B**) Spinal cord; (**C**) Lung; (**D**) Liver; (**E**) Myocardium.

Notably, the SARS-CoV-2 nucleocapsid protein was expressed in multiple organs, as demonstrated by IHC. Of interest is that extensive regions of the brain, spinal cord, lungs, and liver were positively stained for the protein (Figure 8).

In terms of neuronal tissue, numerous SARS-CoV-2 nucleocapsid-positive regions were found in close proximity to and within the germinal matrix of the developing brain (Figure 8A). In the spinal cord, areas positive for the studied protein were observed at the white and grey matter interface. Notably, the paravertebral striated muscle fibers did not express the nucleocapsid marker (Figure 8B).

In the fetal lungs and liver, positive staining for SARS-CoV-2 nucleocapsid protein could be found in perivascular regions, including within cells with morphology indicative for macrophages (Figure 8C,D).

Within the heart, SARS-CoV-2 nucleocapsid positive regions were detected in the myocardial interstitium (Figure 8E).

The number of SARS-CoV-2 nucleocapsid protein positive regions was largest in the brain (2–5/mm^2^), followed by the liver (1–3/mm^2^), lungs (1–2/mm^2^), and myocardium (0–2/mm^2^). Density analysis revealed areas of positive staining and average SARS-CoV-2-positive cell density found in the observed organs. The largest stained areas were observed in the lungs and smallest—in the brain (Table 1).

## 3. Discussion

Inflammation of the placenta inducing acute or chronic placental insufficiency and causing subsequent spontaneous miscarriage or fetal retardation is reported in 40% of maternal infections with Middle East respiratory syndrome coronavirus (MERS-CoV) and severe acute respiratory syndrome coronavirus (SARS-CoV) [7,8]. Additional studies on pregnant women with COVID-19 infection are crucial in order to determine/elucidate whether SARS-CoV-2 has a similar detrimental effect on fetal development.

Vertical transmission of viral infections is a process that depends on a plethora of factors, such as differential viral tropism to the various cell components of the placenta [9]. Current data on vertical transmission of SARS-CoV-2-causing COVID-19 infection remain inconclusive. It has been established that SARS-CoV-2 possesses placental tropism, through ACE2 receptors present predominantly on syncytiotrophoblasts, which allow the virus to enter into these cells and consequently spread through the fetal circulation [10]. Another plausible mechanism of transplacental infection transmission as described for other pathogens such as the cytomegalovirus could be neonatal Fc-receptor-mediated transcytosis into syncytiotrophoblasts, cytotrophoblasts, and macrophages [11].

Of particular interest in our case is the significant quantity of macrophages (Hofbauer cells) in the placenta, especially in the villous stroma, but also in the intervillous space. Normally, these cells have a fetal origin and are a typical stromal component in the chorionic villi, where they first appear on days 10–18 post conception. Their increased number indicates various pregnancy-related pathological conditions, including ascending infections as well as blood transferrable infections causing villous inflammation such as syphilis or cytomegalovirus [12]. Abnormal proliferation of Hofbauer cells is possible during SARS-CoV-2 infection. Similar cellular alterations have been reported in intrauterine Zika virus infection cases [13]. It has been shown that once the Zika virus reaches the chorionic villi, it replicates in Hofbauer cells, thereby spreading from the parietal decidua to the amniochorionic membranes [14]. It is therefore conceivable that macrophages contribute to SARS-CoV-2 reaching the villous vasculature, thereby obtaining access to the fetal circulation.

Moreover, Hofbauer cells seem to play a role in the vertical transmission of HIV. It has been demonstrated that these placenta-specific macrophages express HIV-co-receptor. Genetic variants of this receptor are associated with increased risk of maternal–fetal transmission of the infection [15].

In the present case, inflammatory and necrotic hallmarks in the intervillous space were observed. Damage to placental structures with necrotic features in the syncytiotrophoblast could either be associated with direct cytotoxic effect of SARS-CoV-2 or with circulatory disturbances caused by acute or chronic intervillositis as previously described by other authors [16]. Degradation of the syncytiotrophoblast layer possibly facilitates the spread of the virus in the villous stroma and its subsequent entry into the fetal circulation [16].

In the studied pregnant patient, the observed morphological characteristics of the placenta and amniochorionic membranes clearly indicating an inflammatory process are the most likely cause of the ensuing miscarriage. The fetal circulatory/vascular disturbances—sludge phenomenon and sporadic diapedesis—resemble internal organ alterations resulting from endothelial damage in adult COVID-19 patients. Nonetheless, such anomalies are also characteristic of other conditions, such as ischaemia.

In terms of affected organs, the presence of multiple SARS-CoV-2 nucleocapsid-positive areas close to and in the germinal matrix combined with the notable microcirculatory abnormalities in this brain region could have detrimental consequences for the developing glial and neuronal cells. It is conceivable that this might cause neurodevelopmental alterations after birth, as previously described with vertical Zika virus transmission [17]. It could also lead to the formation of epileptogenic zones, with impaired cortical lamination or other patomorphological changes of the central nervous system.

In the other organs, the majority of SARS-CoV-2 nucleocapsid protein stained cells are likely parenchymal; however, we hypothesize that some of them may be macrophages, especially in the liver (Figure 8D).

Apart from the lack of generalizability of the findings, a limitation of the present study is the inability to exclude other potential causes of the spontaneous miscarriage, such as cervical insufficiency or undiagnosed systemic or local infection. We did not perform a PCR to investigate the fetal blood or amniotic fluid for the presence of SARS-CoV-2.

Recent studies have demonstrated that SARS-CoV-2 virus has neurodevelopmental consequences in newborns with COVID-19 infection, resulting in neuronal damage and neonatal encephalopathy [18,19]. Future research is necessary to evaluate the risk of developing psychiatric disorders, epileptogenic lesions, and other neurologic conditions following SARS-CoV-2 infection. Reports of possible liver damage following vertical transmission of COVID-19 infection exist in the literature [4,20]. Some authors describe abnormal development of the kidneys in cases of COVID-19 infection during the third trimester [21]. Other recent studies report increased heart rate and higher incidence of cardiac anomalies in neonates with COVID-19 infection [4,19,22].

## 4. Conclusions

In conclusion, SARS-CoV-2 infection during the early pregnancy stages could have unexpected consequences for the developing fetus. We present evidence supporting vertical transmission of the virus at the level of the CNS and other vital organs. The potentially detrimental impact of COVID-19 acquired in utero on processes such as cell proliferation, migration, and maturation underlying the development of these organs should be evaluated in future studies. Pronounced placental damage with signs of inflammation and necrosis could explain the cases of miscarriage or stillbirth in pregnant women with active COVID-19 infection, particularly among patients with severe disease course. IHC investigation for SARS-CoV-2 nucleocapsid protein in the fetal organs and placenta could be of benefit for establishing new vertical transmission cases, where the infection might be masked by an immature immune response—a characteristic observation in routine post-mortem examinations of histologically immature tissues of a developing organism.

## Figures and Tables

**Table 1:** Area and enumeration of cells in SARS-CoV2-positive clusters in different fetal organs.

Organ	Average Density of SARS-CoV-2-Positively Stained Regions (N/mm^2^)	Average Area of the SARS-CoV-2-Positive Regions (μm^2^)	Average SARS-CoV-2-Positive Cell Density (Cells/μm^2^)
Brain	3	3257	2.38 × 10^−5^
Myocardium	1	4072	1.55 × 10^−5^
Liver	2	3448	1.58 × 10^−4^
Lungs	1	5792	2.20 × 10^−4^
Average (all organs)	1.75	4129.6	2.00 × 10^−5^

## Data Availability

Not applicable.
